# Application of modified porcine xenograft by collagen coating in the veterinary field: pre-clinical and clinical evaluations

**DOI:** 10.3389/fvets.2024.1373099

**Published:** 2024-03-19

**Authors:** Hyun Min Jo, Kwangsik Jang, Kyung Mi Shim, Chunsik Bae, Jung Bok Park, Seong Soo Kang, Se Eun Kim

**Affiliations:** ^1^Department of Veterinary Surgery, College of Veterinary Medicine and BK21 Plus Project Team, Chonnam National University, Gwangju, Republic of Korea; ^2^Biomaterial R&BD Center, Chonnam National University, Gwangju, Republic of Korea; ^3^MedPark Co., Ltd., Busan, Republic of Korea

**Keywords:** porcine xenograft, collagen coating, hydrophilicity, usability, bone defect, dog

## Abstract

**Introduction:**

This study aimed to identify a collagen-coating method that does not affect the physicochemical properties of bone graft material. Based on this, we developed a collagen-coated porcine xenograft and applied it to dogs to validate its effectiveness.

**Methods:**

Xenografts and collagen were derived from porcine, and the collagen coating was performed through N-ethyl-N’-(3- (dimethylamino)propyl) carbodiimide/N-hydroxysuccinimide (EDC/NHS) activation. The physicochemical characteristics of the developed bone graft material were verified through field emission scanning electron microscope (FE-SEM), brunauer emmett teller (BET), attenuated total reflectance-fourier transform infrared (ATR-FTIR), and water absorption test. Subsequently, the biocompatibility and bone healing effects were assessed using a rat calvarial defect model.

**Results:**

The physicochemical test results confirmed that collagen coating increased bone graft materials’ surface roughness and fluid absorption but did not affect their porous structure. *In vivo* evaluations revealed that collagen coating had no adverse impact on the bone healing effect of bone graft materials. After confirming the biocompatibility and effectiveness, we applied the bone graft materials in two orthopedic cases and one dental case. Notably, successful fracture healing was observed in both orthopedic cases. In the dental case, successful bone regeneration was achieved without any loss of alveolar bone.

**Discussion:**

This study demonstrated that porcine bone graft material promotes bone healing in dogs with its hemostatic and cohesive effects resulting from the collagen coating. Bone graft materials with enhanced biocompatibility through collagen coating are expected to be widely used in veterinary clinical practice.

## 1 Introduction

Bone grafting is a surgical procedure that replaces missing bone with materials from the patient’s body and artificial, synthetic, or natural substitutes (1). Bone grafting is used in various clinical fields to fill bone defects and stimulate new bone formation. Bone fractures are one of the most common ailments in veterinary orthopedics and are typically treated through surgical correction (2). However, in some cases, complications after surgery can lead to improper recovery, resulting in delayed union, malunion, or nonunion of bone (3). Bone grafting is necessary when there is a significant bone union abnormality, or when the bone defect region is too large (4). Bone grafting has recently been employed in veterinary dentistry to fill bony defects (5).

Bone defects in dentistry are primarily caused by trauma, periodontal disease, surgical extraction, cranioplasty, infection, congenital malformation, or oral masses. Among these, tooth loss is the most prevalent (5). Tooth extraction, the most common procedure in veterinary dentistry, inevitably forms bone defects. The alveolar bone is a tooth-dependent tissue. When a tooth is extracted, the alveolar bone, periodontal ligament, and gingiva begin to recover. At this time, alveolar bone regeneration occurs progressively and irreversibly but irregularly (6). According to previous studies, alveolar ridge resorption occurs during the healing process after tooth extraction (7, 8). Specifically, in small or extra-small dogs, the ratio of tooth roots to bone size is high (9). Consequently, the extraction of large teeth, such as canines or molars, increases the risk of fracture due to the limited remaining mandibular alveolar bone. Research on bone grafting in the dental field has recently surged to prevent this problem.

Biological mechanisms such as osteoinduction, osteoconduction, and osteogenesis are essential for facilitating proper bone healing. Thus, various bone graft materials are utilized to support these processes (10, 11). Bone graft materials usually have one or more components: an osteoconductive matrix to support new bone ingrowth, osteoinductive proteins to support mitogenesis of undifferentiated cells, and osteogenic cells (osteoblasts or osteoblast precursors) to form bone in the proper environment (11). In addition, materials that promote bone formation, such as membranes or recombinant human bone morphogenetic protein-2 (rhBMP-2), are continuously researched to improve bone grafting efficacy (12, 13).

Generally, bone graft materials can be categorized into autograft, allograft, xenograft, and synthetic bone graft substitutes, each with advantages and disadvantages (11, 14). Autografts, which are biocompatible and have high osteogenic potential, are considered the gold standard for bone transplantation. However, some drawbacks include additional surgery, risks of infection, and limited amounts of graft material that can be harvested. Therefore, allograft or xenograft materials are often used as alternative options (15, 16).

Xenografts, like allografts, are one of the most commonly used methods for bone grafting. This method holds a distinct advantage over allografts due to its larger production scale at a lower cost (17). Xenografts using bovine bone were the first to be developed, and various bone graft materials have been crafted since, such as Bio-Oss® (Geistlich Biomaterials, Wolhusen, Switzerland) or Cerabone® (Botiss Biomaterials GmbH, Zossen, Germany). In recent human medicine, research has been conducted on bone graft materials derived from porcine bones instead of bovine, as they have tissue and organ compositions similar to humans (17, 18).

Possible post-bone grafting side effects include graft material absorption, graft material dislodgement, graft site infection, and potential damage to neighboring or adjacent anatomical structures, such as the neurovascular bundle (19). Bone graft dislodgement is a common side effect; dislodged graft materials can prompt such as ectopic bone formation (20). If excessive bleeding occurs during bone grafting, applying graft material may be difficult, and bone graft materials may dislodge after surgery. Therefore, forming a mechanical barrier using an absorbable or non-absorbable membrane on the graft material is used to prevent these risks (21). However, the surgical process is arduous; non-absorbable membranes require secondary removal surgery, and absorbable membranes have an uncontrolled barrier function duration because it is challenging to control the absorption rate (22, 23).

Collagen is found in large quantities in porcine skin and has hemostatic and cohesive effects that promote angiogenesis and epithelial tissue regeneration (24). Due to this effect, methods of mixing collagen components with various bone graft materials have recently been continuously studied (25). In particular, several experimental studies have investigated mixing various bovine-derived bone graft materials or synthetic bone graft substitutes with collagen (12, 21, 26). Nevertheless, there has been relatively limited research on combining collagen with bone graft material derived from porcine bone (14), and its clinical applications are rare in veterinary medicine. Therefore, this study conducted collagen coating to enhance the hemostatic and cohesive effects of porcine-derived bone graft materials. This method was expected to facilitate the manipulation of bone graft material during surgery and prevent the migration of graft material after surgery. Additionally, we evaluated the developed xenograft’s biocompatibility and applied it in orthopedic and dental cases to confirm its bone-healing effect.

## 2 Materials and methods

### 2.1 Bone graft

The bone graft material (Particle size: 0.3–0.5 mm, Bone-D XP, MedPark, Busan, Korea, Cat# MBXP-P021-015) and medical-grade collagen, derived from porcine bone and skin, respectively, were produced by MedPark Co., Ltd. The collagen coating process on the bone graft material was executed through the following procedure. A collagen solution was prepared by stirring porcine-derived collagen in acetic acid for 8 h until complete dissolution. Then, we intended a reaction between the hydroxyl group (OH-) present on the bone graft material’s surface and the coupling agent 3-aminopropyltriethoxymethoxysilane (3-APTES). As a result, this process formed amino groups (-NH_2_) on the bone graft material’s surface. These amino groups (-NH_2_) and the collagen’s carboxyl group (-COOH) are covalently bonded though N-ethyl-N′-(3-(dimethylamino)propyl) carbodiimide/N-hydroxysuccinimide (EDC/NHS) activation. This step facilitates a strong adhesion between collagen and the bone graft material, a process known as collagen immobilization of bone graft materials. After mixing and immersing the surface-treated bone graft material with the collagen solution, only the bone graft material was filtered. Subsequently, the material was washed several times with purified water, followed by freeze-drying. Finally, all bone graft materials used in the experiment were provided by Medpark (Busan, Korea) after being sterilized through gamma irradiation.

### 2.2 Evaluation of physicochemical properties

#### 2.2.1 Field emission scanning electron microscope

All collagen-coated bone grafts were observed through FE-SEM (Hitachi, Ltd., Tokyo, Japan) to detect morphological features on the particle’s surface.

#### 2.2.2 Brunauer–Emmett–Teller

The Korea Institute of Ceramic Engineering & Technology (Jinju-si, KICET) was requested to perform the BET test. The surface area and porosity of the collagen-coated bone grafts were measured following the KS L ISO 18757 guideline.

#### 2.2.3 Attenuated total reflectance-Fourier transform infrared

Samples were analyzed through ATR-FTIR (PerkinElmer, Inc., Waltham, MA, United States) to confirm the chemical characteristics on the surface. ATR spectra were recorded at a 4 cm^−1^ resolution, and the recording range was 4,000–450 cm^−1^.

#### 2.2.4 Water absorption test

The water absorption test was conducted on each bone graft material (*n* = 3) in phosphate-buffered saline (PBS, Thermo Fisher Scientific INC. Korea, Seoul, Korea, Cat# 14040133) at pH 7.4 and 37°C. Initially, the weight of the bone graft materials (*W_dry_*) was measured and immersed in a PBS solution. After intervals of 1, 4, and 24 h, the bone graft materials were removed from the PBS solution. Excess PBS was removed using filter paper (Whatman® qualitative filter paper, Merck Ltd. Korea, Seoul, Korea, Cat# WHA10010155), and the weight (*W_wet_*) was measured.


$$\mathrm{Swelling ratio}(\%)=\frac{W_{\mathrm{wet}}-W_{\mathrm{dry}}}{W_{\mathrm{dry}}}\text{X}100$$

### 2.3 *In vivo* evaluation in a rat calvarial defect model

#### 2.3.1 Experimental animals

The Institutional Animal Care and Use Committee of Chonnam National University in Korea approved this animal study (Approval No. CNU IACUC-YB-2020-93). This study included 40 healthy 7-week-old male Sprague–Dawley rats (weight: 300–320 g; Samtaco, Osan, Korea). The housing environment was an air-conditioned room with a controlled temperature of 23 ± 2°C, a 12-h light–dark cycle, and a relative humidity of 60 ± 10%. The rats were randomly provided tap water and a commercial rodent diet (Samyang Feed Co., Ltd., Incheon, Korea). The animals were randomly allocated into four groups (Control group: only critical defect; non-coated group: porcine-derived bone graft; 0.5% collagen-coated group: porcine-derived bone graft coated with 0.5% collagen; 0.75% collagen-coated group: porcine-derived bone graft coated with 0.75% collagen).

#### 2.3.2 Anesthesia and surgical procedure

General anesthesia was performed with 10 mg/kg xylazine (Bayerkorea Ltd., Seoul, Korea) and 100 mg/kg ketamine (Yuhan Co., Seoul, Korea) through intraperitoneal injection. Pain was controlled using 20 mg/kg tramadol through intraperitoneal injection (Huons Co., Seongnam, Korea). The skin of the rat’s head was disinfected and incised to expose the periosteum of the skull. A calvarial defect (diameter: 8 mm) was made using an 8 mm trephine bur and a surgical micromotor (NSK, Tokyo, Japan). Next, 0.03 g of collagen-coated bone grafts were implanted into the created defect. The periosteum was then sutured with 4–0 Surgisorb® (Samyang Co., Seongnam, Korea), and the skin was closed with 3–0 Black silk® (Ailee Co., Ltd., Busan, Korea). Animals were euthanized using CO_2_ 4 and 8 weeks post-operation.

#### 2.3.3 Microcomputed tomography 3D analysis

Micro-CT was used to analyze samples at 130 kVp and 60 μA radiation levels with a SkyScan 1173 Desktop X-ray microtomograph (SkyScan; Bruker-CT, Kontich, Belgium). Measurements were taken with SkyScan1173 control software (Ver. 1.6, Bruker-CT, Kontich, Belgium) under the following conditions: 130 kVp tube voltage, 60 μA tube current, and a 1 mm aluminum filter. Next, 800 high-resolution images were obtained with an exposure time of 500 ms, 2,240 × 2,240 pixels, 13.88 μm pixel size, 0.3° rotation angle, and 180° rotation. Section reconstruction was performed using Nrecon (Ver 1.7.4.6, Bruker-CT), and the axis of the section image was arranged using Dataviewer (Ver. 1.5.6.2, Bruker-CT, Kontich, Belgium). The Ct Analyzer (Ver. 1.19.4.0, Bruker-CT, Kontich, Belgium) was used to analyze the new bone volume at the defect site. The region of interest (ROI) was to avoid invading the host bone as much as possible. Grayscale values ranging from 68 to 255 represented mineralized tissue, with the range of 68–99 indicating newly mineralized tissue within the defects. Values between 100 and 255 were considered representative of the bone graft materials. The new bone volume (NBV, mm^3^) was the sum of newly formed bone volumes in the defect.

#### 2.3.4 Histological evaluation

First, samples were fixed using 10% buffered formalin for 24 h, and the specimens were decalcified with Calci-Clear™ Rapid (National Diagnostics, Atlanta, GA, United States, Cat# HS-105) for 1 week. Subsequently, an ascending series of alcohol rinses were used for sample dehydration, and specimens were embedded in paraplast (Sherwood Medical Industries, Deland, FL, USA). Embedded samples were sectioned to a 5 μm thickness with a microtome (Cambridge Instruments, Germany), and slides were stained with hematoxylin and eosin (H&E), Masson-Goldner trichrome (GT), and Van Gieson’s (VG) for microscopic observation (27, 28). In the VG microscopic images, the new bone area was measured for quantitative analysis with the Image J program (National Institute of Health, Stapleton, NY, United States).

#### 2.3.5 Statistical analysis

All data represent the mean ± standard error of the mean. A one-way ANOVA analysis and Tukey’s *post hoc* test were conducted with a statistical program (GraphPad Prism 8.0 software; GraphPad Software, Inc., Boston, MA, United States) to evaluate the new bone volume in micro-CT and histological images. *p* values <0.05 were considered statistically significant.

### 2.4 Clinical cases

The study included two orthopedic and one dental problem in dogs. Owner consent for including patient data in this clinical study was obtained in all cases.

#### 2.4.1 Case 1

**Figure 1:**
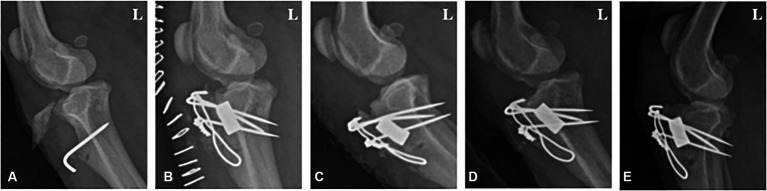
Radiographic image of a 1-year-old male poodle with avulsion fractures of the tibial tuberosity. **(A)** Lateral radiograph displaying a nonunion fracture of the tibia before surgery. **(B)** 1, **(C)** 2, **(D)** 4, and **(E)** 8 weeks post-operation. L: Left.

A 1-year-old, 6.3 kg male poodle was referred to Chonnam National University—Veterinary Medical Teaching Hospital (CNU-VMTH) for left tibial tuberosity exposure. The patient was diagnosed with left medial patellar luxation (MPL) and underwent surgery, including tibial tuberosity transposition, at a local animal hospital. However, a gap between the tibial tuberosity and tibia was observed 1 week after surgery, and a second surgery was performed. Ten days after the second surgery, a surgical wound dehiscence occurred, exposing the tibial tuberosity. Subsequently, the patient was admitted to CNU-VMTH for treatment. Through radiographic images, we confirmed the exposed tibial fragment and surgical pin, and a gap between the tibial tuberosity and tibia was observed, with no evidence of bone union (Figure 1A). Therefore, after repositioning the exposed tibia to its normal position, a collagen-coated bone graft application was planned to fill the gap between the tibial tuberosity and the tibia.

Before surgery, the patient intravenously received 0.5 mg/kg famotidine (Gaster INJ 20 mg; Dong-a Pharm, Seoul, Korea) and 20 mg/kg cefazolin (Cefazolin CKD INJ 1 g; Chong Kun Dang Pharm, Seoul, Korea). Then, the patient was premedicated with 0.005 mg/kg glycopyrrolate (Glycopyrrolate Reyon AMP 1 mL; Reyon Pharm, Seoul, Korea) subcutaneously, followed by 0.3 mg/kg morphine (Morphine HCl INJ 10 mg/mL; Hana Pharm, Seoul, Korea) and 5 μg/kg medetomidine (Tomidin 1 mg/mL; Provet Veterinary Products Ltd., Istanbul, Turkey) through intramuscular injection. Anesthesia was induced intravenously using 2 mg/kg propofol (Provive INJ 1%, 10 mg/mL; Myungmoon Pharm, Seoul, Korea). Inhalation anesthesia was maintained with 2% isoflurane (Forane®; JW Pharmaceutical, Seoul, Korea) in 100% oxygen administered via an endotracheal tube.

After removing the skin and subcutaneous sutures from the latest surgery, a lateral incision was made on the stifle joint to expose the fracture site. The tibial avulsion fracture site was identified, and the Kirschner wire that was applied was removed. Tibial tuberosity was trimmed, repositioned, and fixed with a Kirschner wire using the tension band wiring technique. The gap between the tibia and tibial tuberosity was filled with a collagen-coated bone graft loaded with rhBMP-2 (Cowellmedi Co., Busan, Korea). Postoperatively, the patient received 0.5 mg/kg famotidine (Gaster INJ 20 mg; Dong-a, Seoul, Korea), 12.5 mg/kg amoxicillin hydrate/diluted potassium clavulanate (Amocla INJ 0.6 g; Kuhnil Pharm, Seoul, Korea), and 2.2 mg/kg carprofen (Rimadyl injectable 50 mg/mL; Zoetis Korea Ltd., Seoul, Korea) intravenously for 3 days. Then, 0.5 mg/kg famotidine (Famotidine Tab. 20 mg Nelson; Nelson, Chungbuk, Korea), 12.5 mg/kg amoxicillin hydrate/diluted potassium clavulanate (Lactamox Tab amoxicillin 50 mg/tab; clavulanate 125 mg/tab; Aprogen pharm, Sungnam, Korea), and 2.2 mg/kg carprofen (2.2 mg/kg, Rimadyl Tab 25 mg/tab; Zoetis Korea Ltd., Seoul, Korea) were orally administered twice per day for 7 days. A soft padded bandage was maintained for 4 weeks after surgery to stabilize the surgical site.

#### 2.4.2 Case 2

**Figure 2:**
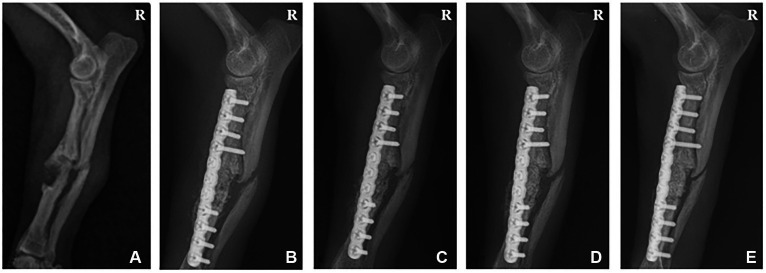
Radiographic image of a 6-year-old female chihuahua with radioulnar fracture. **(A)** Lateral radiograph displaying a simple fracture of the radius and ulna before surgery. **(B)** 1, **(C)** 2, **(D)** 4, and **(E)** 6 weeks post-operation. R: Right.

A 6-year-old, 1.8 kg female Chihuahua was referred to CNU-VMTH for a right radioulnar fracture. The patient sustained a complicated fracture of the right radius and ulnar 3 years prior and received surgery using a plate and screw system at a local animal hospital. However, bone-crushing occurred when inserting the screw, so a tension bend wiring technique was applied together. Eight months after surgery, the plate, screws, and wire were removed. However, the fracture did not heal completely, and re-fracture occurred. Radiographs revealed a simple fracture on the middle part of the right radius and ulnar and a hypertrophic nonunion around the fracture site (Figure 2A). Therefore, the radius was rearranged properly through a plate and screw system. Next, we planned to fill the interfragmentary gap using a collagen-coated bone graft to promote bone regeneration.

The same anesthetic protocol in Case 1 was used, excluding medetomidine. Instead, 0.3 mg/kg midazolam (Midacum INJ 1 mg/mL; Myungmoon Pharm, Seoul, Korea) was intravenously injected in place of medetomidine.

A lateral incision to the left radius exposed the fracture site. The radioulnar fracture site was confirmed, and the radius was trimmed because of malunion at the fracture site. For accelerate the bone healing, we made the microfracture on the radius bone marrow cavity by Kirschner wire. The fracture was stabilized using a Ø1.2 plate and screw system. The gap between the bone fragments was filled using 0.25 mg rhBMP-2-loaded (Cowellmedi Co., Busan, Korea) collagen-coated bone graft. Then, the collagen membrane (Lyoplant; B. Braun, Melsungen, Germany) was applied to prevent bone graft displacement from the graft site.

Postoperative medications from Case 1 were replicated. A soft padded bandage was applied to ensure stability at the surgical site for 6 weeks following surgery.

#### 2.4.3 Case 3

A 2-year-old, 3.6 kg mixed-breed neutered male dog was referred to CNU-VMTH for left mandibular edema. A local animal hospital diagnosed a dentigerous cyst on the left mandibular region. The patient’s overall health and appetite were normal, but swelling in the mandible continued to increase in size. Radiographs were taken for an accurate assessment, revealing an impacted canine tooth in the alveolar bone. Therefore, we planned for canine tooth extraction, and a collagen-coated bone graft was used on the extraction defect to support alveolar bone healing.

The anesthetic protocol used in Case 1 was replicated. For local anesthesia, 1 mg/kg bupivacaine (Bupivacaine HCl INJ 0.5% 1 mg/mL; Myungmoon Pharm, Seoul, Korea) was administered, and an additional analgesic effect was achieved through a constant rate infusion of ketamine (Yuhan Ketamine 50 INJ, 50 mg/mL; Yuhan, Seoul, Korea) at 10 μg/kg/min.

**Figure 3:**
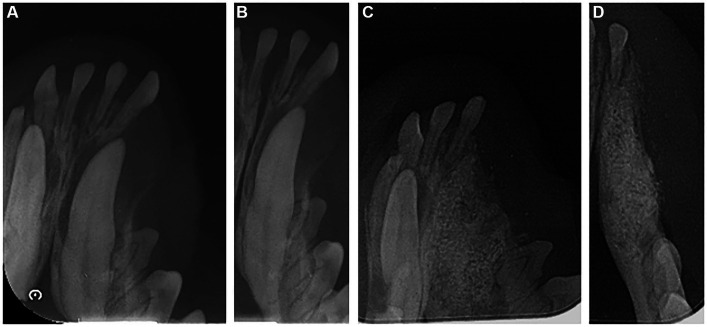
Radiographic image of the left mandible of a 2-year-old mixed neutered male dog. **(A,B)** Dental radiograph displaying an impacted canine tooth in the alveolar bone before extraction. **(C,D)** 6 months post-operation.

After anesthesia, dental scaling and x-rays were taken, and the dental radiograph indicated an impacted canine tooth in the left mandible (Figures 3A, B). A gingival incision was used to expose the canine tooth. An alveolectomy was performed around the impacted canine tooth, and the tooth was extracted. A collagen-coated bone graft filled the alveolar bone defects following extraction, and a collagen membrane was applied to prevent dislodging.

Postoperative medications were the same as those in Case 1. Subsequently, we advised the client to stick to wet food feedings for 2 weeks after surgery.

## 3 Results

### 3.1 Physicochemical property evaluation

**Figure 4:**
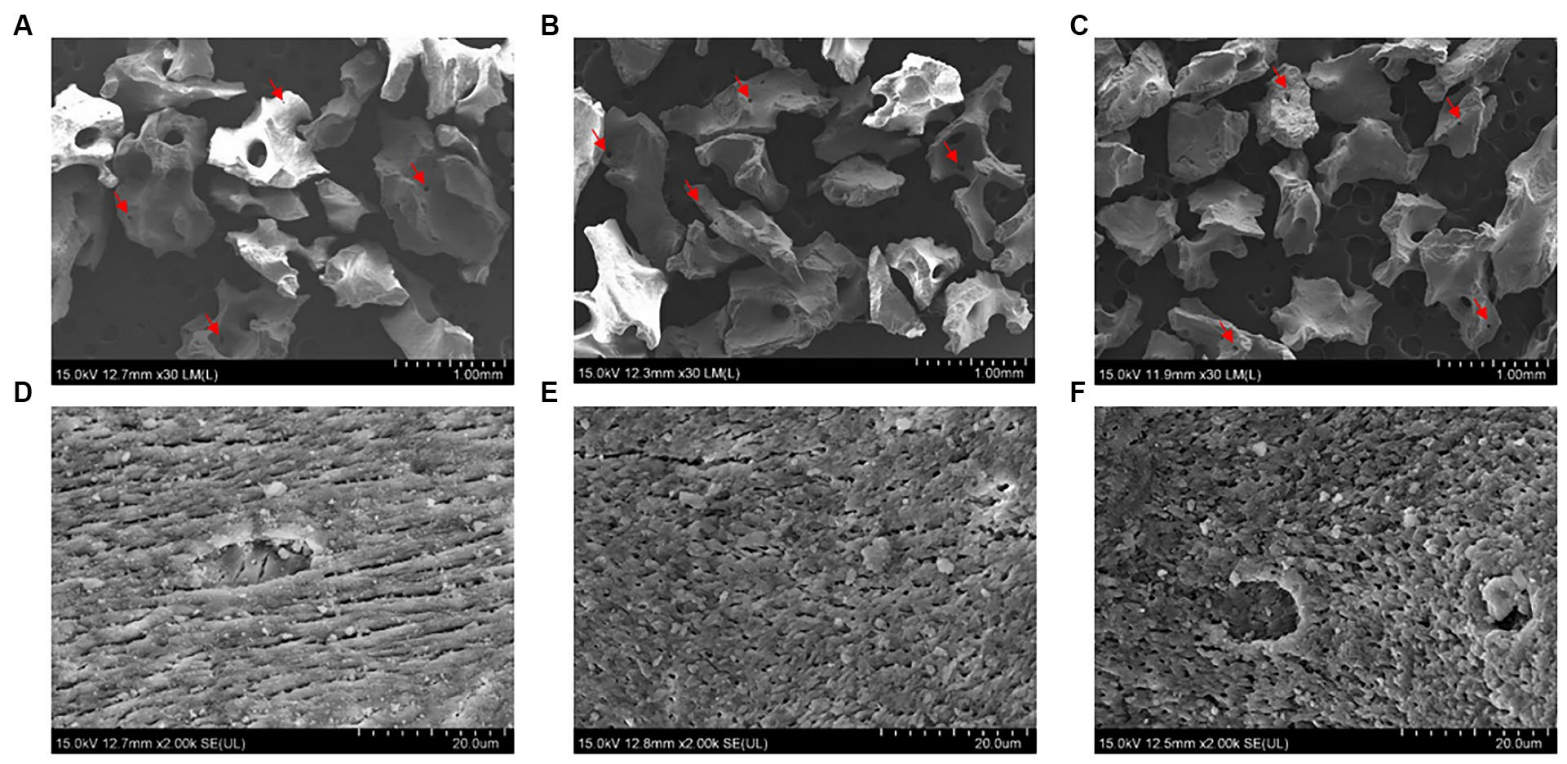
Scanning electron microscope (SEM) images of bone graft material surfaces. Low magnification (30×) of **(A)** non-coated, **(B)** 0.5% collagen-coated, and **(C)** 0.75% collagen-coated bone graft material. High magnification (2,000×) of **(D)** non-coated, **(E)** 0.5% collagen-coated, and **(F)** 0.75% collagen-coated bone graft material. Red arrows: pores.

We conducted FE-SEM, BET, ATR-FTIR, and water absorption tests to evaluate the physiochemical properties of the collagen-coated bone graft materials. The FE-SEM images of all bone graft materials revealed heterogeneous structures and a similar particle size of around 1 mm. Regardless of whether the collagen was coated, a macropore was observed on the surface of all graft materials (Figures 4A–C). In the high-magnification images, a rough surface was observed for all bone graft materials. The roughness increased in the following order: non-coated group, 0.5% collagen-coated group, and 0.75% collagen-coated group. In addition, the porous structure was well-maintained in all groups (Figures 4D–F).

**Figure 5:**
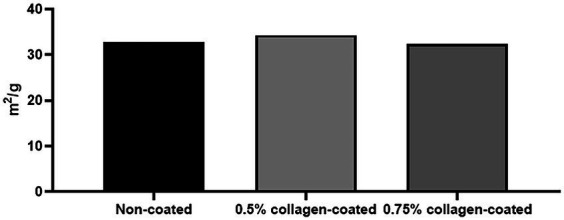
Brunauer Emmer Teller (BET) surface area data for collagen-coated bone grafts. BET surface area results following gas adsorption confirmed that the porous structure of the collagen-coated bone graft materials were similar to non-coated bone graft materials.

**Figure 6:**
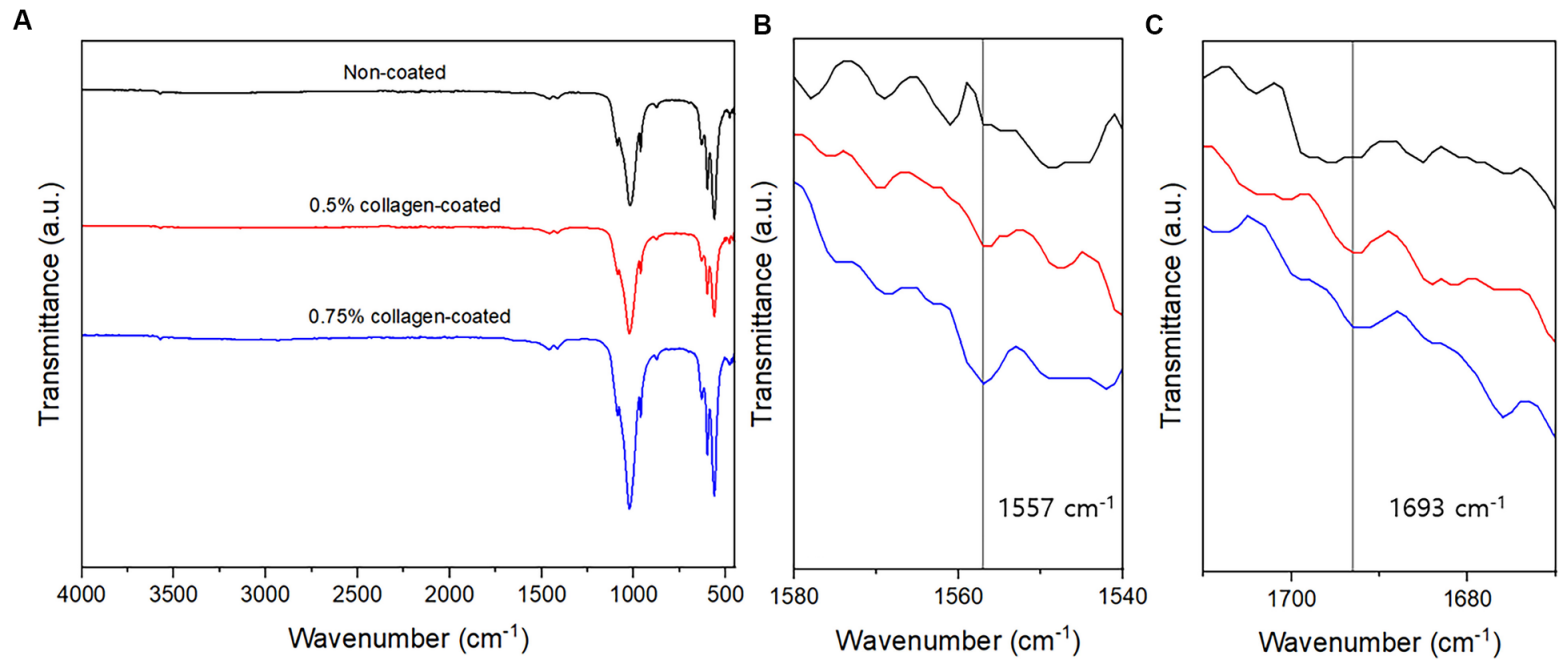
Attenuated total reflectance-Fourier transform infrared (ATR-FTIR) of bone graft materials. **(A)** ATR-FTIR spectra between 500 and 4,000 cm^−1^; **(B)** At a 1,540–1,580 cm^−1^ magnification, the specific peaks characteristic of collagen were observed at 1,557 cm^−1^; **(C)** At a 1,670–1710 cm^−1^ magnification, the specific peaks characteristic of collagen were observed at 1,693 cm^−1^.

**Figure 7:**
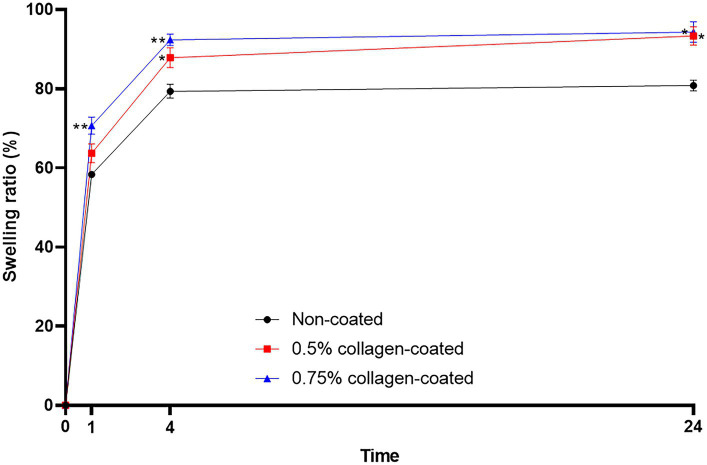
Water absorption test of bone graft materials. The swelling ratio of 0.75 collagen-coated bone graft material exhibited a notable increase compared to the non-coated graft material. Furthermore, the collagen-coated group displayed a significantly higher swelling ratio than the non-coated group at 4 and 24 h (*p* < 0.05).

The BET surface area of the 0.5 and 0.75% collagen-coated bone graft materials were 34.4 m^2^/g and 32.4 m^2^/g, displaying a similar porous structure, unlike those of the non-coated bone graft material (32.9 m^2^/g) (Figure 5). All bone graft materials were analyzed through ATR-FTIR (Figure 6). All groups shared similar values overall; however, collagen’s typical bands at 1693 cm^−1^ for amideI and 1,557 cm^−1^ for amideII were observed in both collagen-coated groups. During the water absorption test, the 0.75% collagen-coated bone graft materials exhibited higher absorbability than the non-coated graft material after 1 h (*p* < 0.01). After 4 and 24 h, the collagen-coated group demonstrated significantly enhanced absorbability compared to the non-coated group (*p* < 0.05) (Figure 7).

### 3.2 *In vivo* evaluation with rat calvarial defect model

#### 3.2.1 Micro-CT 3D analysis

**Figure 8:**
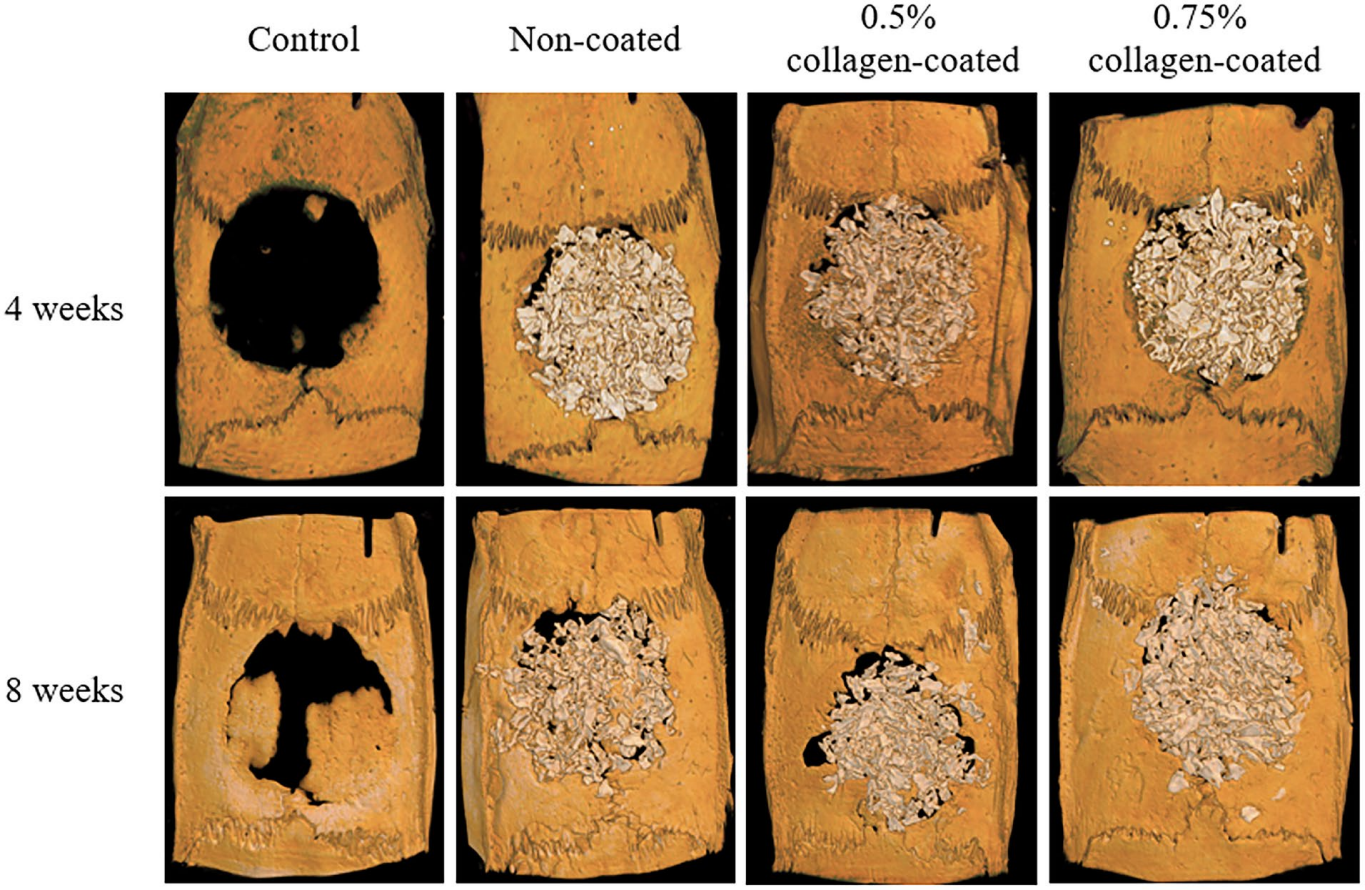
Micro-computed tomography (Micro-CT) three-dimensional (3D) images of rat calvarial defects 4 and 8 weeks after bone grafting.

**Figure 9:**
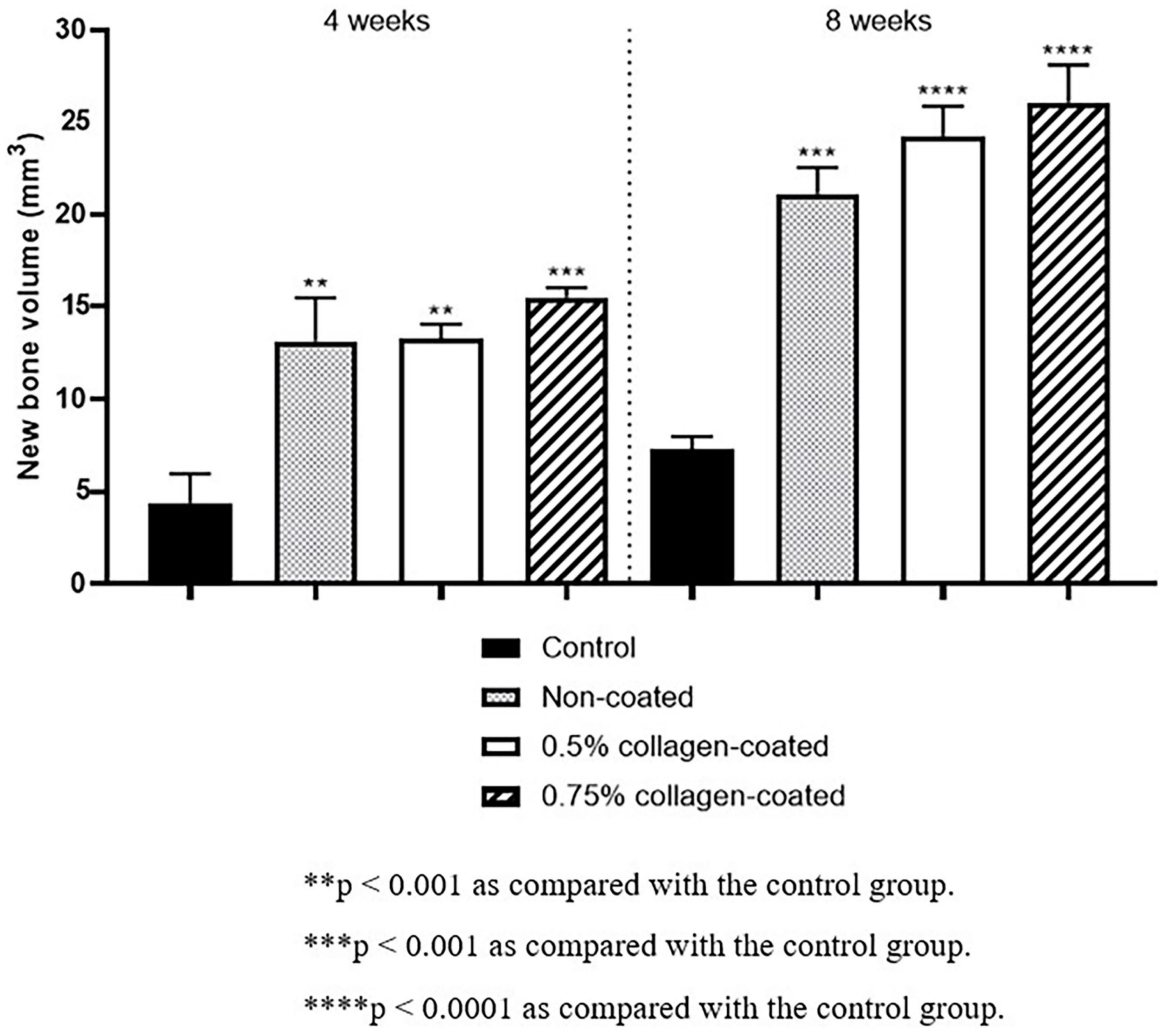
New bone volumes of rat calvarial defects 4 and 8 weeks after bone grafting using Micro-computed tomography (Micro-CT).

The Micro-CT 3D analysis revealed that all groups exhibited new bone formation from the defect margin. At 4 and 8 weeks, bone grafts were well-maintained in the defect area across all experimental groups (Figure 8). At 4 weeks after the grafting, the NBV of all bone-grafted groups was significantly higher than the control group (*p* < 0.01), and no difference was observed by collagen concentrations. The NBV of all collagen-coated bone grafts increased significantly more at 8 weeks than the control group (*p* < 0.001), and no difference was observed according to the collagen concentrations (Figure 9).

#### 3.2.2 Histological evaluation

**Figure 10:**
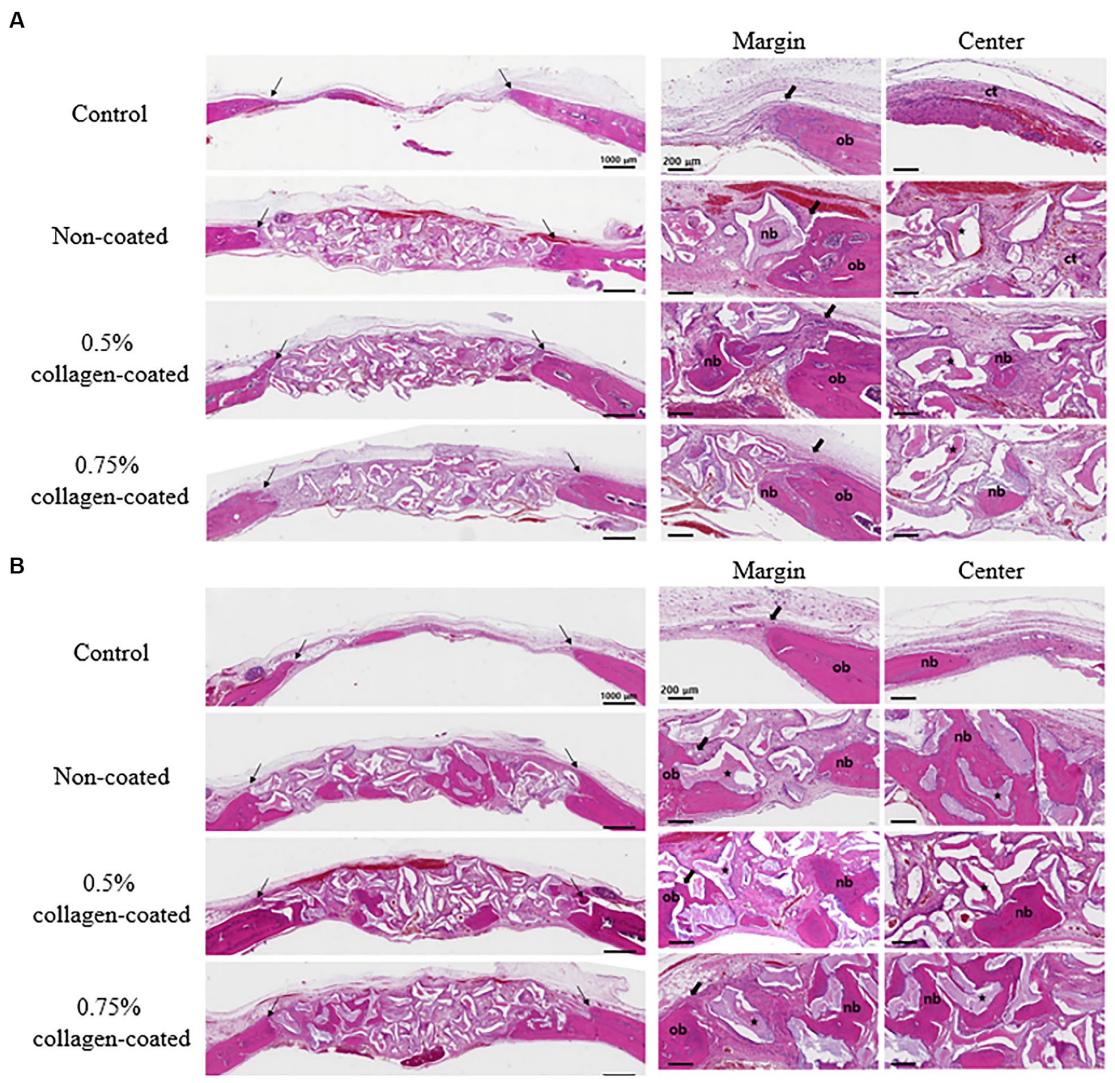
Hematoxylin Eosin staining results of calvarial defects in rats 4 and 8 weeks after bone grafting. **(A)** 4 weeks after bone grafting. **(B)** 8 weeks after bone grafting. Control group: only critical defects. Black arrows: defect margin; ct: connective tissue; nb: new bone; ob: old bone; star: bone graft materials.

**Figure 11:**
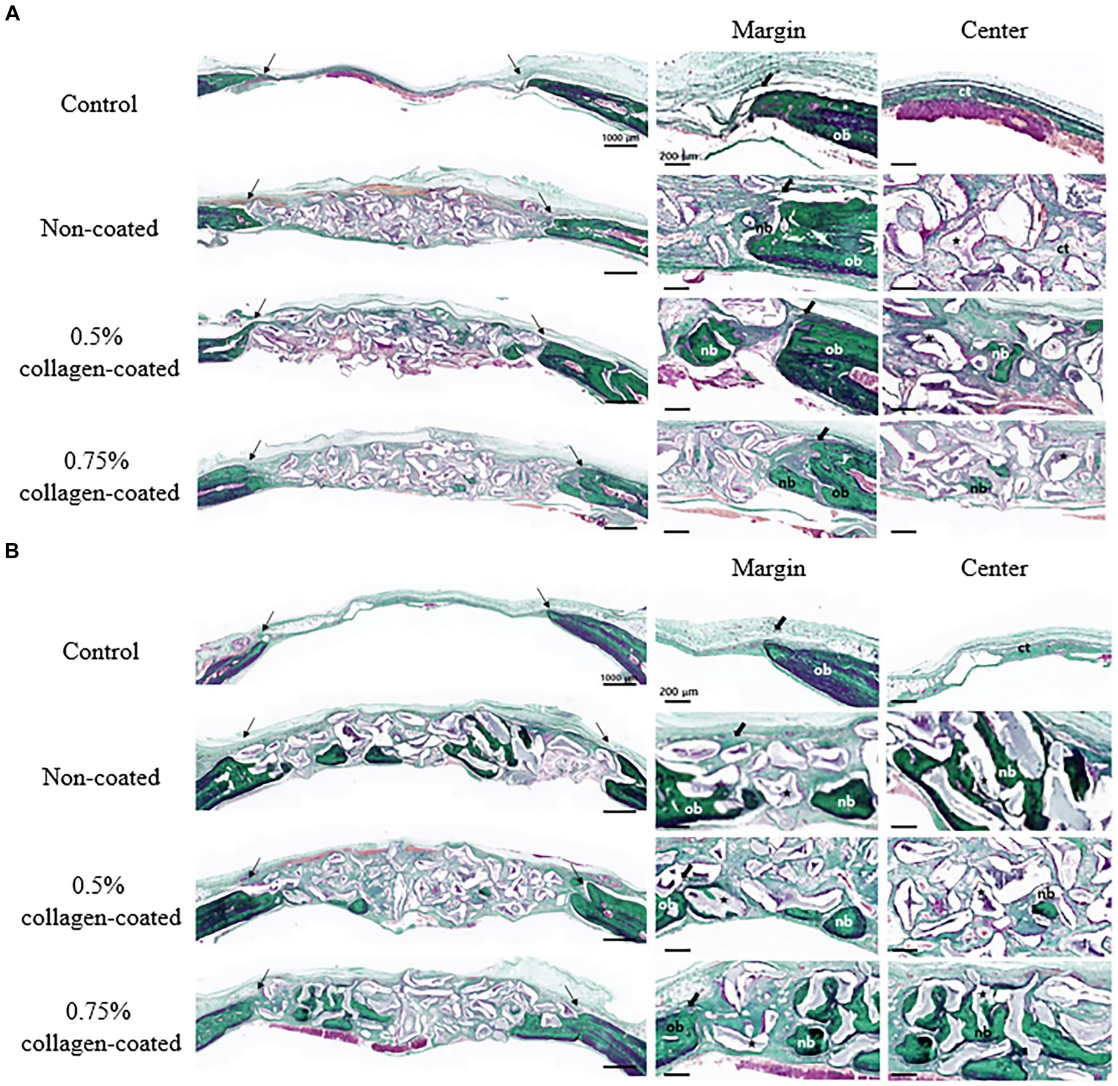
Masson-Goldner trichrome staining results of calvarial defects in rats 4 and 8 weeks after bone grafting. **(A)** 4 weeks after bone grafting. **(B)** 8 weeks after bone grafting. Control group: only critical defects. Black arrows: defect margin; ct: connective tissue; nb: new bone; ob: old bone; star: bone graft materials.

**Figure 12:**
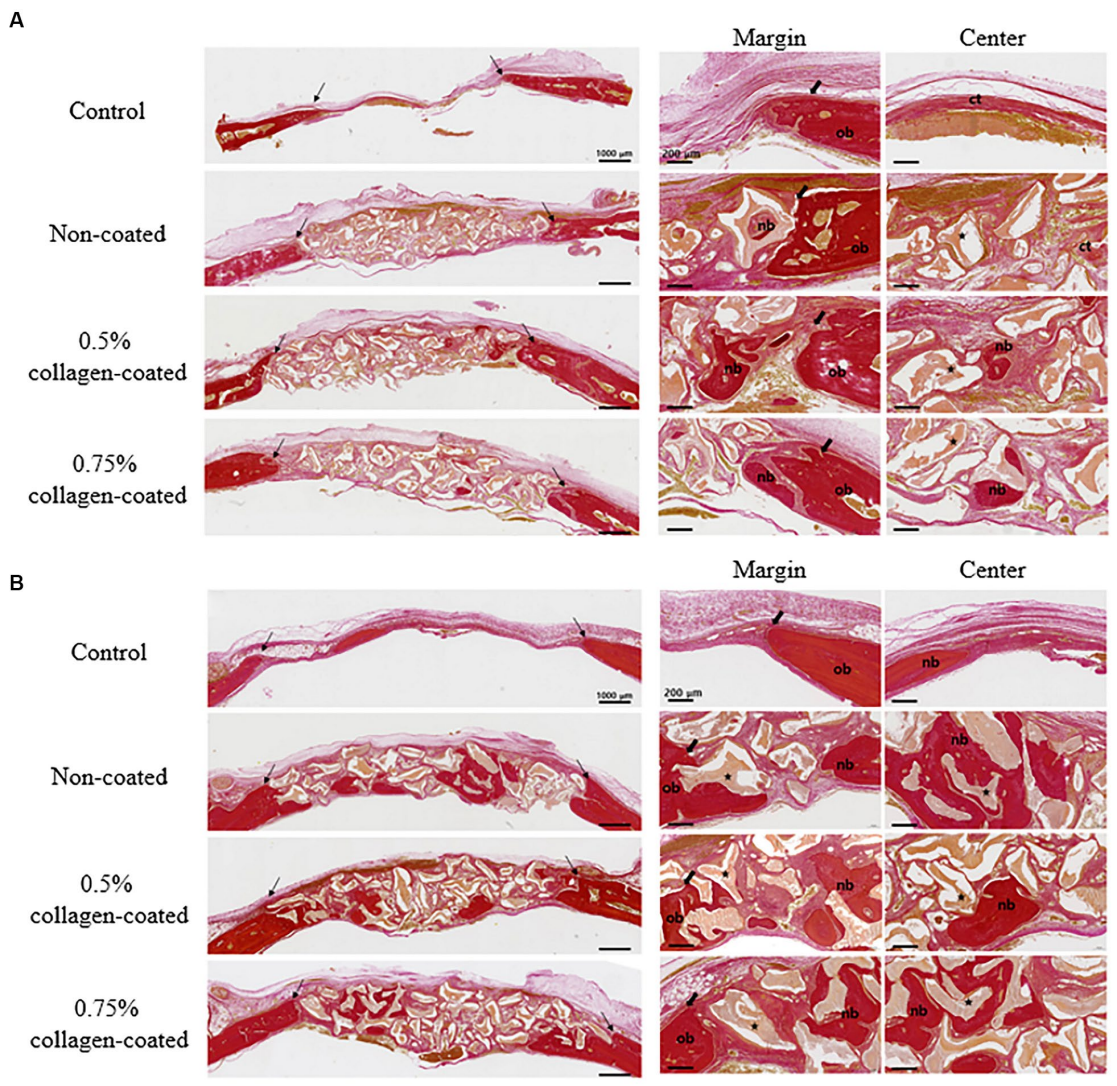
Van Gieson’s staining results of calvarial defects in rats 4 and 8 weeks after bone grafting. **(A)** 4 weeks after bone grafting. **(B)** 8 weeks after bone grafting. Control group: only critical defects; Black arrows: defect margin; ct: connective tissue; nb: new bone; ob: old bone; Star: bone graft materials.

In the H&E histological images, no experimental group exhibited an adverse reaction, such as inflammation, at the bone graft site 4 and 8 weeks after implantation (Figure 9). In the control group, fibrous connective tissues in the margin and defect center area were observed 4 weeks post-implantation, but new bone formation was not. However, new bone formation with fibrous connective tissues was confirmed in all bone graft groups’ margin and defect center regions, regardless of the collagen percentage (Figures 10A, 11A, 12A). At 8 weeks post-implantation, minor bone regeneration was confirmed in the defect’s center but not in the margin in the control group. Regardless of the collagen-coated, new bone formed in all bone graft groups at the margin and the defect’s center. In particular, more new bone filled the center of the defect area than at 4 weeks (Figures 10B, 11B, 12B).

**Figure 13:**
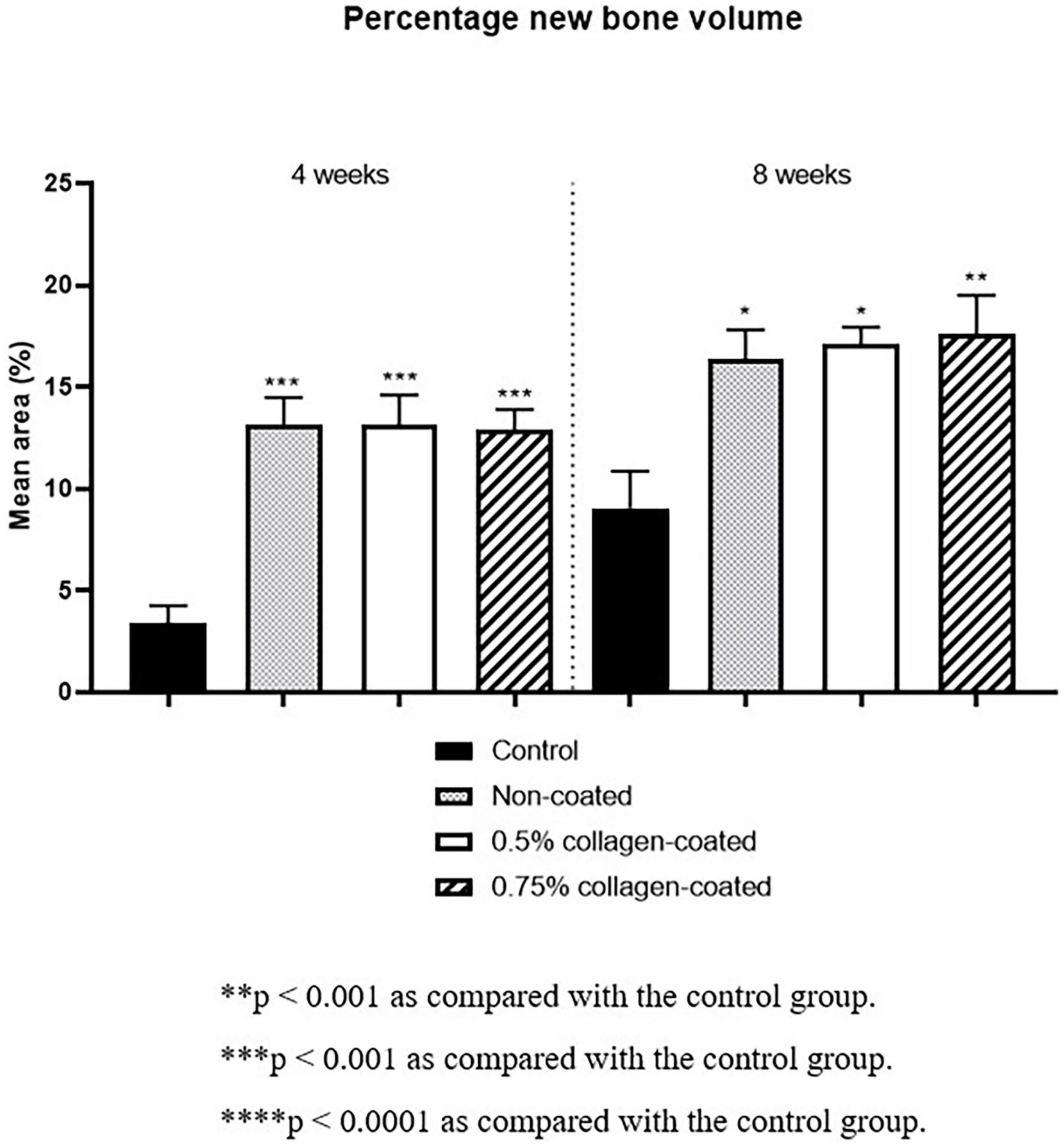
New bone areas of rat calvarial defects 4 and 8 weeks after bone grafting in Van Gieson’s stained images. Control group: only critical defects.

Moreover, in the analysis of the VG-stained histological images 4 weeks post-implantation, the new bone area in all graft groups was significantly higher than in the control group (*p* < 0.001), with no differences in collagen concentration. By the eighth week, the new bone area in all graft groups had significantly increased compared to the control group (*p* < 0.05), while no disparities in collagen concentration were noted (Figure 13).

### 3.3 Clinical cases

#### 3.3.1 Case 1

Follow-up radiographic imaging was conducted 1, 2, 4, and 8 weeks after surgery. The bone alignment appeared well-maintained in the radiographs taken 1 and 2 weeks after surgery; however, a fracture line was observed between the tibial tuberosity and the tibia (Figures 1B,C). The fracture line was faintly visible in the radiographs 4 weeks post-surgery (Figure 1D). Bone union was completed, and the fracture line was no longer visible 8 weeks post-surgery (Figure 1E).

#### 3.3.2 Case 2

Follow-up radiographic imaging was performed 1, 2, 4, and 6 weeks after surgery. The plate and screw were well-maintained on the radiographs 1 and 2 weeks after surgery. However, the fracture line was visible, and the bone graft site density was lower than the normal bone (Figures 2B,C). Radiographs 4 weeks post-surgery indicated that the bone graft site density was similar to normal bone, and the fracture gap was narrowed (Figure 2D). In the radiographs 6 weeks after surgery, the fracture site was completely recovered and no longer visible (Figure 2E).

#### 3.3.3 Case 3

Dental radiography was performed 6 months post-surgery. These images revealed that the extraction defect in the canine region had been successfully filled with new bone, and the new bone’s density resembled that of the surrounding alveolar bone (Figures 3C,D).

## 4 Discussion

In veterinary orthopedics and dentistry, bone healing can be enhanced by selecting an appropriate bone graft material for fractures with large bone defects or delayed fracture healing. The choice of bone graft material affects the outcome of the surgery, and various bone graft materials are constantly being developed to meet these requirements (29). Recently, research has been conducted on the advantages and disadvantages of the raw materials of bone grafts and the graft material’s shape and size (30, 31). Especially when dealing with particle-type bone graft materials, securing them in the correct location can occasionally prove challenging. This is primarily due to the presence of bleeding, which often exposes a significant challenge for surgeons during the surgical procedure. Therefore, considering a bone graft material’s effectiveness in promoting bone formation and its ease of manipulation, which can significantly impact the operation’s success.

Previous studies enhanced bone graft materials by combining them with various substances, such as platelet-rich fibrin and polydeoxyribonucleotide. However, the former must be obtained directly from the patient, and the latter requires additional mixing immediately before transplantation, adding an extra step to the process (32, 33). Furthermore, these methods are primarily aimed at improving the function of bone grafting. In contrast, large quantities of collagen exist in pig skin, so it can be easily extracted and pre-mix with graft material, simplifying its use. In addition, a collagen coating on porcine-derived bone graft materials with its hemostatic and cohesive effects was developed to enhance bone recovery and facilitate operation. We crafted 0.3–0.5 mm particle-sized bone graft materials from porcine bones. Subsequently, collagen coating was applied to the surface of these bone graft materials.

Because collagen is biocompatible, biodegradable, easily available, and highly versatile (34), many coating methods on various implants (such as dental implants, mesh, and 3D printed scaffolds) are being studied (35–37). Typically, this coating is achieved by combining collagen and implants through immersion in a mixture (38). Bone grafting materials and collagen mixtures are also undergoing research; however, similar to other implants, bone grafting materials and collagen are often used by simple mixing (26). In these methods, where the bone graft material and collagen are not completely combined, the resulting stability is low, and achieving uniformity is challenging due to the inherent nature of the mixture. Conversely, the collagen coating method used in this study chemically treats the bone graft material’s surface. This process involves covalently bonds the collagen molecule’s carboxyl group with the bone graft material’s amino group using EDC/NHS activation. This method enables more stable and uniform bonding than simple mixing.

We conducted several analyses, including FE-SEM, BET, and ATR-FTIR, to assess the physicochemical properties of the collagen-coated bone graft material. Initially, FE-SEM imaging confirmed the surface and porous structure of the bone graft material. We discovered that collagen coating increases surface roughness to a degree that does not affect the material’s porosity. Notably, the surface roughness of the bone graft material affects protein absorption, promoting platelet adhesion and activation, ultimately influencing bone healing (39). Additionally, the implant’s porosity plays a critical role in the bone healing process. When the porous structure is obstructed, it can impede the formation of new blood vessels and vital tissues, potentially delaying bone recovery (40). Therefore, when combining bone graft material with other substances, it is essential to verify that the porous structure remains unaltered.

Brunauer emmett teller was employed to quantify a solid sample’s specific surface area and pore size distribution by adsorbing and desorbing a gas on its surface (41) to assess the bone graft material’s specific surface area quantitatively. This test indicated no difference in surface area between the collagen-coated and non-coated groups. The SEM and BET test results confirmed that the graft material’s pore distribution remained unchanged after collagen coating. The porous structure plays a crucial role in promoting new bone formation by facilitating vascular transport, nutrient delivery, and removal of cellular waste (40). According to the above research results presented, collagen-coated bone graft materials exhibit the ability to form new bone through a porous structure, similar to non-coated bone grafts.

The wavenumbers associated with collagen’s vibrational frequencies were detected through ATR-FTIR analysis to verify the collagen coating on the bone graft materials. Amide I (1,600–1,800 cm^−1^) and amide II (1,470–1,570 cm^−1^) collagen-specific wavenumber bands are well-known IR characteristics of amides (42). In our results, peaks were observed within the typical wavenumber range associated with amide I and amide II, indicating collagen on the bone graft material’s surface. Furthermore, collagen-coated bone graft materials exhibited notably high swelling ratios during the water absorption test. This finding suggests that the collagen-coated bone graft material can absorb biological fluids, such as blood, at a faster and higher rate than non-coated bone graft materials. The high swelling capabilities of collagen-coated bone graft materials provide hemostatic and cohesive effects by concentrating clotting factors within the sample (43, 44).

Based on the above results, we established a method that successfully attached collagen at 0.5 and 0.75% concentration to the bone graft material’s surface. While this attachment increased surface roughness, it did not affect the material’s physical structure, such as porosity. Through this process, we developed a high-absorbency graft material by coating it with collagen, a protein commonly lost during deproteinization when producing xenografts through traditional chemical and heat treatments.

Next, an animal experiment was conducted using a rat calvarial defect model to evaluate the biocompatibility and bone healing efficacy of collagen-coated bone graft material. The micro-CT scans revealed that all bone graft groups exhibited more substantial bone healing than the control group at 4 and 8 weeks post-implantation. However, there was no discernible correlation between collagen coating and the extent of bone healing. Histological assessments further confirmed the superior rate of new bone formation in all bone graft groups compared to the control group. Notably, similar to the micro-CT findings, there was no observed correlation between collagen coating and bone recovery level.

Minor new bone formation was observed in the control group 8 weeks after post-implantation. Comparatively, the bone graft groups were observed throughout the experimental period, with new bone formation confirmed around the graft material. Regardless of whether the bone graft materials were collagen-coated, they were gradually degraded and absorbed throughout the experiment. Furthermore, no side effects, such as inflammation, were observed in the surrounding tissues. This finding substantiates that applying collagen-coated bone graft material *in vivo* does not induce adverse reactions and that the collagen coating does not influence the bone recovery capabilities of bone graft materials.

We assessed the practical applicability and bone recovery potential using 0.75% collagen-coated bone graft material in clinical scenarios. The first case exhibited an avulsion fracture between the tibial tuberosity and the tibia from a complication following medial patellar luxation surgery. Bone union was repeatedly delayed even after re-operation at a local hospital. A gap between the fragments remained even after the correction, so bone grafting was conducted to fill the gap. Next, a combination of the trimmed bone fragments and collagen-coated bone grafts was mixed with rhBMP-2 to preclude additional surgical procedures. Upon follow-up radiography, a faint fracture line was observed 4 weeks after surgery, and complete union was confirmed after 8 weeks.

The second case involved a re-fracture following the removal of the implant due to insufficient bone healing after surgery. After correcting the fractured bone, the same graft material combination in Case 1 was used. In the postoperative radiological follow-up, the graft area exhibited a density similar to the surrounding bone 4 weeks post-surgery. Eight weeks after the surgery, a complete union was confirmed, with no fracture line was detected, and normal limb function was restored. When fractures repeatedly occur, filling the gaps between bone fragments is essential to ensure proper alignment correction and promote normal bone healing. Filling the gap between bone fragments with bone grafting material provides mechanical stability during healing, leading to appropriate fracture reduction.

Collagen-coated bone grafts and rhBMP-2 were used in these two cases because the dog was too small to yield enough graft material to fill the fracture gap. Since the osteoinductive properties of xenografts were lost due to the chemical and thermal treatments, rhBMP-2 was added for osteoinduction. rhBMP-2 possesses potent osteoinductive capabilities, promoting chondrogenesis, osteogenesis, angiogenesis, and regulation of extracellular matrix synthesis in animals and humans. In general, the bone recovery process commences around 7 weeks after surgery. In contrast, we confirmed that bone healing in our cases was faster than the expected bone regeneration process. In particular, as a carrier, collagen efficiently absorbs and releases drugs such as antibiotics, nonsteroidal anti-inflammatory drugs, steroids, and rhBMP-2 solutions well (45–47). This characteristic suggests that collagen-coated bone grafts may promote bone formation faster than in other cases.

In the last case, bone grafting was performed on the bony defect after a tooth extraction, and a collagen-coated bone graft was applied to promote alveolar bone recovery. A radiograph taken 6 months after surgery confirmed the alveolar bone recovery in the extraction socket. Normally, additional bone grafting is not required for jaw cystic lesions (48). However, recent research has focused on the effect of bone regeneration through bone grafting after removing the cystic lesion (49, 50). Additionally, the tooth roots-to-bone size ratio is notably high in small breed dogs, especially regarding mandibular teeth, resulting in a small amount of alveolar bone after extraction. Consequently, this increases the risk of jaw fractures. Therefore, we applied collagen-coated bone grafts to fill the defect area after tooth extraction with cystectomy. The height of the alveolar bone tends to decrease during bone recovery following tooth extraction (51); however, in our case, alveolar bone recovery was successful without alveolar bone loss.

In conclusion, the physicochemical evaluation results confirm that various concentrations of collagen coating do not affect the physical properties of bone graft materials. Moreover, the coating enhances operator convenience through hemostatic and cohesive effects and successfully promotes bone recovery when applied in orthopedics and dentistry. This study has two limitations. Firstly, the non-clinical trial involved various histologic analyses commonly employed in bone regeneration studies, but it lacked an analysis of the expression and distribution of bone regeneration proteins, such as osteocalcin and alkaline phosphatase, through techniques like immunohistochemical staining or ELISA. Secondly, the restricted number of clinical cases limits the ability to definitively determine the effectiveness of the bone graft material in clinical applications. These limitations underscore the need for further supplementation through future studies. Additionally, future research should consider whether collagen-coated bone graft material can perform as a vehicle for drug delivery utilizing collagen’s absorption ability. If so, these studies are expected to develop a new type of clinically significant bone graft material.

## Data availability statement

The original contributions presented in the study are included in the article/supplementary material, further inquiries can be directed to the corresponding authors.

## Ethics statement

The animal studies were approved by Institutional Animal Care and Use Committee of Chonnam National University in Korea (Approval No. CNU IACUC-YB-2020-93). The studies were conducted in accordance with the local legislation and institutional requirements. Written informed consent was obtained from the owners for the participation of their animals in this study.

## Author contributions

HJ: Conceptualization, Data curation, Formal analysis, Investigation, Methodology, Validation, Visualization, Writing – original draft, Writing – review & editing. KJ: Methodology, Validation, Visualization, Writing – original draft, Conceptualization, Data curation, Writing – review & editing. KS: Validation, Writing – review & editing. CB: Validation, Writing – review & editing. JP: Writing – review & editing, Resources. SSK: Supervision, Validation, Writing – review & editing. SEK: Conceptualization, Methodology, Supervision, Validation, Writing – review & editing.
